# Supplementary material for: FAST RESENSITIZATION OF G PROTEIN-COUPLED RECEPTORS REQUIRES THEIR PI(4,5)P2-DEPENDENT SORTING INTO AN AP2 POSITIVE COMPARTMENT
Source: bioRxiv. 2025 Apr 1:2025.03.28.645988. Preprint. [Version 1] doi: 10.1101/2025.03.28.645988 (PMC12190482; doi:10.1101/2025.03.28.645988)

bioRxiv preprint doi: https://doi.org/10.1101/2025.03.28.645988; this version posted April 1, 2025. The copyright holder for this preprint (which was not certified by peer review) is the author/funder. This article is a US Government work. It is not subject to copyright under 17 USC 105 and is also made available for use under a CC0 license.

LEGENDS TO SUPPLEMENTARY FIGURES

Figure S1. Monitoring PM PPIns levels in resting and stimulated cells after knocking-down distinct PIP5K1A and EFR3 isoforms

(A) Representative Western Blot analysis of HEK-AT1 lysates from cells treated with the indicated isoform specific siRNA against PIP5K1 proteins.

(B) PM PI4P and **(C)** PI(4,5)P_2 levels in unstimulated HEK-AT1 cells after silencing of distinct PIP5K1 isoforms. Basal PM PPIns levels were assessed by the resting BRET-Ratio values obtained before stimulation in each experiment in cells expressing the PI4P and PI(4,5)P_2 BRET sensors. Data are means ± SEM of three **(B)** or four **(C)** independent experiments, each performed in triplicates. Statistical difference was calculated using one-way ANOVA followed by Tukey's post-hoc test. (n.s: not significant)

(D) Monitoring changes in PM PI4P levels after M1R activation in HEK-AT1 cells knocked down for the indicated PIP5K1A isoforms (control – blue, PIP5K1A – red, PIP5K1B – green, PIP5K1C – purple) using a BRET analysis. After a 5 min control period, cells were treated with 100 µM carbachol (CCh) followed by the addition of 10 µM atropine after 15 mins. Bar graphs show area under the curve (AUC) calculations for the BRET values covering the recovery (atropine) period to evaluate statistical differences between the groups. Data are means ± SEM of three independent experiments, each performed in triplicates. Scatter plots show results of individual experiments. Statistical significance was obtained by using one-way ANOVA followed by Tukey post-hoc test to estimate differences between the separate groups in multiple comparisons. (n.s: not significant)

(E) Monitoring changes in PM PI4P levels after AT1R activation in HEK-AT1 cells treated with the indicated targeting siRNAs. Cells were stimulated with 100 nM AngII after a 5 min control period. Data are means ± SEM of three independent experiments, each performed in triplicates. Columns show mean ± SEM of AUC calculations scatters representing the individual values. One-way ANOVA followed by Tukey's post-hoc test was used for evaluation of the statistical differences in multiple comparisons. Blue asterisks display the differences compared to the control, while black asterisks represent

bioRxiv preprint doi: https://doi.org/10.1101/2025.03.28.645988; this version posted April 1, 2025. The copyright holder for this preprint (which was not certified by peer review) is the author/funder. This article is a US Government work. It is not subject to copyright under 17 USC 105 and is also made available for use under a CC0 license.

differences between the different treatment groups (n.s.: non-significant; *: $p<0.05$; **: $p < 0.01$).

(F-G) Comparisons of single or double knockdown of PIP5K1A and EFR3 isoforms on the activity of **(E)** AT1R or **(F)** β-adrenergic receptors (βAR) using BRET-based measurement of PM PI(4,5)P_2 or cytoplasmic cAMP levels, respectively, in HEK-AT1R cells treated with the indicated siRNA(s). Cells were stimulated with 100 nM AngII to stimulate AT1R **(F)** or with 3µM isoprenaline (Iso) **(F)** to stimulate endogenously βARs after a 5 min control period. Bar graphs show area under the curve (AUC) calculations for the curves in both experiments to compare the separate groups. Scatter plots show the results of individual experiments. Data are means ± SEM of **(F)** three or **(G)** four independent experiments, each performed in triplicates. Statistical differences were evaluated by one-way ANOVA followed by Tukey post-hoc test to estimate differences between the separate groups in multiple comparisons. Blue asterisks display the differences compared to the control, while black asterisks represent differences between the different treatment groups. (n.s.: non-significant; *: $p<0.05$; **: $p < 0.01$)

(H-I) Bar graphs show area under the curve (AUC) **(H)** or rate constants (Tau) **(I)** calculations on curves presented in Figure 2G. Scatter plots show the data points from the individual experiments. Data are means ± SEM of five independent experiments, each performed in triplicates. Statistical significance was evaluated by one-way ANOVA followed by Tukey post-hoc test to estimate differences between the separate groups in multiple comparisons. Blue asterisks refer to differences compared to the control, while black asterisks refer to statistical differences between the indicated groups. (n.s.: non-significant; **: $p < 0.01$). Note that while there is a difference in the AUC values in dynamin 2 knockdowns, due to the smaller PI(4,5)P_2 decrease in this group, but the τ value (i.e. rate of desensitization) shows no difference.

(J) Bar graphs show area under the curve (AUC) calculations performed on the data shown in Figure 2H. Cells were treated with the indicated siRNAs and transfected with either empty vector (e.v.) or dominant negative Dynamin2 (K44A). Scatter plots show the results of individual experiments. Data are means ± SEM of seven (control siRNA + e.v. – *blue* and control siRNA + K44A – *red*), five (PIP5K1A siRNA + e.v. – *green* and PIP5K1A

bioRxiv preprint doi: https://doi.org/10.1101/2025.03.28.645988; this version posted April 1, 2025. The copyright holder for this preprint (which was not certified by peer review) is the author/funder. This article is a US Government work. It is not subject to copyright under 17 USC 105 and is also made available for use under a CC0 license.

siRNA + K44A – *magenta*) or four (EFR3A siRNA + e.v. – *orange* and EFR3A siRNA + K44A – *black*) independent experiments, each performed in triplicates. Statistical significance was evaluated by one-way ANOVA followed by Tukey post-hoc test to estimate differences between the separate groups in multiple comparisons. Blue asterisks refer to the difference compared to the control siRNA + e.v. group, while black asterisks refer to comparisons withing the indicated groups (n.s.: non-significant; ***: $p < 0.001$, ****: $p < 0.0001$).

Figure S2. Effects of PIP5K1A or EFR3A knock-down on the PM recruitment of β-arrestins, or clustering of AT1Rs as well as the role of β-arrestin1 on AT1R desensitization

(A-C) Bar graphs showing area under the curve (AUC) calculations on data presented in Figure 3 D-F. Cells were treated with the indicated siRNAs and transfected with either TK-promoter driven β-arrestin1-mVenus **(A)**; βarrestin2-mVenus **(B)**; or they stably expressed AT1R-GFP **(C)**. Scatter plots show the results of individual experiments. Data are means ± SEM of 38 (control siRNA – blue), 37 (PIP5K1A siRNA – red) and 31 (EFR3A siRNA – green) cells in panel **A**; 51 (control siRNA – blue), 43 (PIP5K1A siRNA – red) and 54 (EFR3A siRNA – green) cells in panel **B**, obtained in seven independent experiments. For panel **C** the numbers are: 52 (control siRNA – blue), 39 (PIP5K1A siRNA – red) and 46 (EFR3A siRNA – green) from five independent experiments. Statistical significance was evaluated by one-way ANOVA followed by Tukey post-hoc test to estimate differences between the separate groups in multiple comparisons. (n.s.: non-significant; *: $p < 0.05$, **: $p < 0.01$; ****: $p < 0.0001$)

(D) Rescue experiment performed on HEK-AT1 cells silenced for β-arrestin1 (ARRB1 si) using siRNA-pools and subsequently transfected with TK-promoter driven siRNA-resistant mutants of β-arrestin1. BRET measurements of PM $PI(4,5)P_2$ changes after stimulation with 100 nM AngII are shown. (control siRNA – blue, ARRB1 siRNA – red, control siRNA + TK-βarrestin1 – green, ARRB1 siRNA + TK-βarrestin1 – purple). Bar graphs show area under the curve (AUC) calculations to evaluate statistical differences between the groups. Scatter plots show the results of individual experiments. Data are

bioRxiv preprint doi: https://doi.org/10.1101/2025.03.28.645988; this version posted April 1, 2025. The copyright holder for this preprint (which was not certified by peer review) is the author/funder. This article is a US Government work. It is not subject to copyright under 17 USC 105 and is also made available for use under a CC0 license.

means ± SEM of four independent experiments, each performed in triplicates. Statistical significance was evaluated by two-way ANOVA followed by Tukey post-hoc test to estimate differences between the separate treatment groups in multiple comparisons. Blue asterisks refer to differences compared to control, while black asterisks refer to differences between the indicated groups (n.s.: non-significant; ***: $p < 0.001$).

(E) Representative Western Blots showing the levels of β-arrestin1 and -2 from cell lysates treated with different βarrestin1 specific siRNAs alone or in combination. The upper band is β-arrestin1, the lower one is β-arrestin2.

(F) BRET measurements of AngII-induced PM PI(4,5)P_2 changes measured in HEK-AT1 cells treated with different β-arrestin1 targeting siRNAs. After siRNA treatment (4 days in total), cells were transfected with the BRET-based PI(4,5)P_2 -sensor and stimulated with 100 nM AngII after 5 mins. Data are means ± SEM of three independent experiments, each performed in triplicates.

(G) Representative Western Blots showing the levels of β-arrestin1 and -2 from cell lysates obtained from wild type (WT), β-arrestin 1/2 double knockout, or β-arrestin1 single knockout clones. The upper band shows β-arrestin1 and the lower band represents β-arrestin2.

(H) BRET measurements of AngII-induced PM PI(4,5)P_2 changes measured in HEK-AT1 clones of β-arrestin1 knockouts. Data are means ± SEM of three independent experiments, each performed in triplicates.

(I) Bar graphs showing area under the curve (AUC) calculations on the curves shown in panel H. To statistically compare the different groups, one-way ANOVA followed by Dunnett's multiple comparison was used. Scatter plots show the results of individual experiments. Data are means ± SEM of three independent experiments, each performed in triplicates. Significance levels relate to comparison to WT cells (n.s.: non-significant; *: $p < 0.05$; **: $p < 0.01$; ***: $p < 0.001$).

Figure S3*.* AP2 is required for sustained AT1R activity by supporting βarrestin-1 function

bioRxiv preprint doi: https://doi.org/10.1101/2025.03.28.645988; this version posted April 1, 2025. The copyright holder for this preprint (which was not certified by peer review) is the author/funder. This article is a US Government work. It is not subject to copyright under 17 USC 105 and is also made available for use under a CC0 license.

(A-C) Bar graphs show area under the curve (AUC) or recovery rate constant (τ) calculations performed on the data shown in ***Figure 4 B-D***. Scatter plots show the results of individual experiments. Data are means ± SEM of five **(A-B)** or four **(C)** (except for the control n=5, and SH3GL2 n=3) independent experiments, each performed in triplicates. Statistical significance was evaluated by one-way ANOVA followed by Tukey post-hoc test to estimate differences between the separate groups in multiple comparisons. Blue asterisks refer to differences compared to control, while black asterisks refer to differences between the indicated individual groups (n.s.: non-significant; **: $p < 0.01$; ****: $p < 0.0001$.

(D-G) BRET analysis of PM PI(4,5)P2 changes in AngII stimulated HEK-AT1 cells after knock-down of PIP5K1A **(D)**, EFR3A **(E)**, β-arrestin1 **(F)**, or AP2M1 **(G)** either alone or in combination with silencing β-arrestin2. Data are means ± SEM of six (control siRNA and ARRB2 siRNA), four (PIP5K1A siRNA, EFR3A siRNA, ARRB1 siRNA, EFR3A + ARRB2 siRNAs, AP2M1 + ARRB2 siRNAs) or three (PIP5K1A + ARRB2 and ARRB1 + ARRB2 siRNAs) independent experiments, each performed in triplicates.

Please note, that for comparison, the same control siRNA treated curves are shown in each panel. Also, ARRB1 siRNA-treated trace in panel ***F*** *is also the same shown in Figure 3G.*

Additional Supplementary Data:

βarrestin1 siRNA-resistant sequence:

(NheI)gctagc*acgcgcacc***atgggcgacaaagggacacgagtgttcaagaaggcaagccccaacgggaaactgacagtgtacc
tgggaaagcgggactttgtggaccacattgacctggtggaccccgtggatggcgtggtcctggtggatcctgagtatctcaaagaaa
ggcgagtctacgtgacactgacctgcgccttccggtatggccgggaagacctggatgtcttgggtctgacttttcgcaaagacctgttt
gtggctaacgtgcagtccttcccaccggcccctgaggacaagaagccactgactcggctacaagagcgactcatcaagaagctgg
gcgagcatgcctacccttcacctttgagatcccgccaaaccttccgtgctcagtcacattgcaacctgggcctgaggacacaggg
aaggcctgcggtgtggattatgaagtgaaagccttctgtgctgagaacctggaggagaagatccacaaaaggaattctgtgcggcta
gtcatccggaaggttcaatatgcccctgagaggcctggccctcagcccacggctgagaccaccagacagttcctcatgtcggaca
agcccctgcaccttgaggcatctctagataaagaaatttactatcatggagaacccatcagcgtcaatgtccatgtcaccaacaaca
ccaacaagactgtgaagaagatcaagatctcggtgcgccagtatgcagacatctgtctcttcaacacagctcagtacaagtgccca
gtggccatggaggaagctgatgatactgtggcacccagctcaacattctgcaaggtctacacactgactcccttcctggcaaacaac
agagagaagcgggggcttgccctcgacgggaagctcaagcatgaagacacaaatctggcttccagcactctgttgcgggaaggc
gccaaccgtgaaatcctgggtatcattgtttcctacaaagtcaaagtgaagctggtggtgtcccggggcggcctgttgggagaccttg
catccagtgacgtggctgtggagcttcctttcactctcatgcaccccaagcctaaagaggagcccccacatcgggaagttccagaat
ctgaaaccccagtggacaccaatctcatagagcttgacaccaatgatgacgacattgtgtttgaggactttgctcgtcagcggctgaa
aggcatgaaggatgacaaggacgaagaggatgatggcaccggctctccacacctcaacaacagatag**gcggccgc(NotI)

bioRxiv preprint doi: https://doi.org/10.1101/2025.03.28.645988; this version posted April 1, 2025. The copyright holder for this preprint (which was not certified by peer review) is the author/funder. This article is a US Government work. It is not subject to copyright under 17 USC 105 and is also made available for use under a CC0 license.

bioRxiv preprint doi: https://doi.org/10.1101/2025.03.28.645988; this version posted April 1, 2025. The copyright holder for this preprint (which was not certified by peer review) is the author/funder. This article is a US Government work. It is not subject to copyright under 17 USC 105 and is also made available for use under a CC0 license.

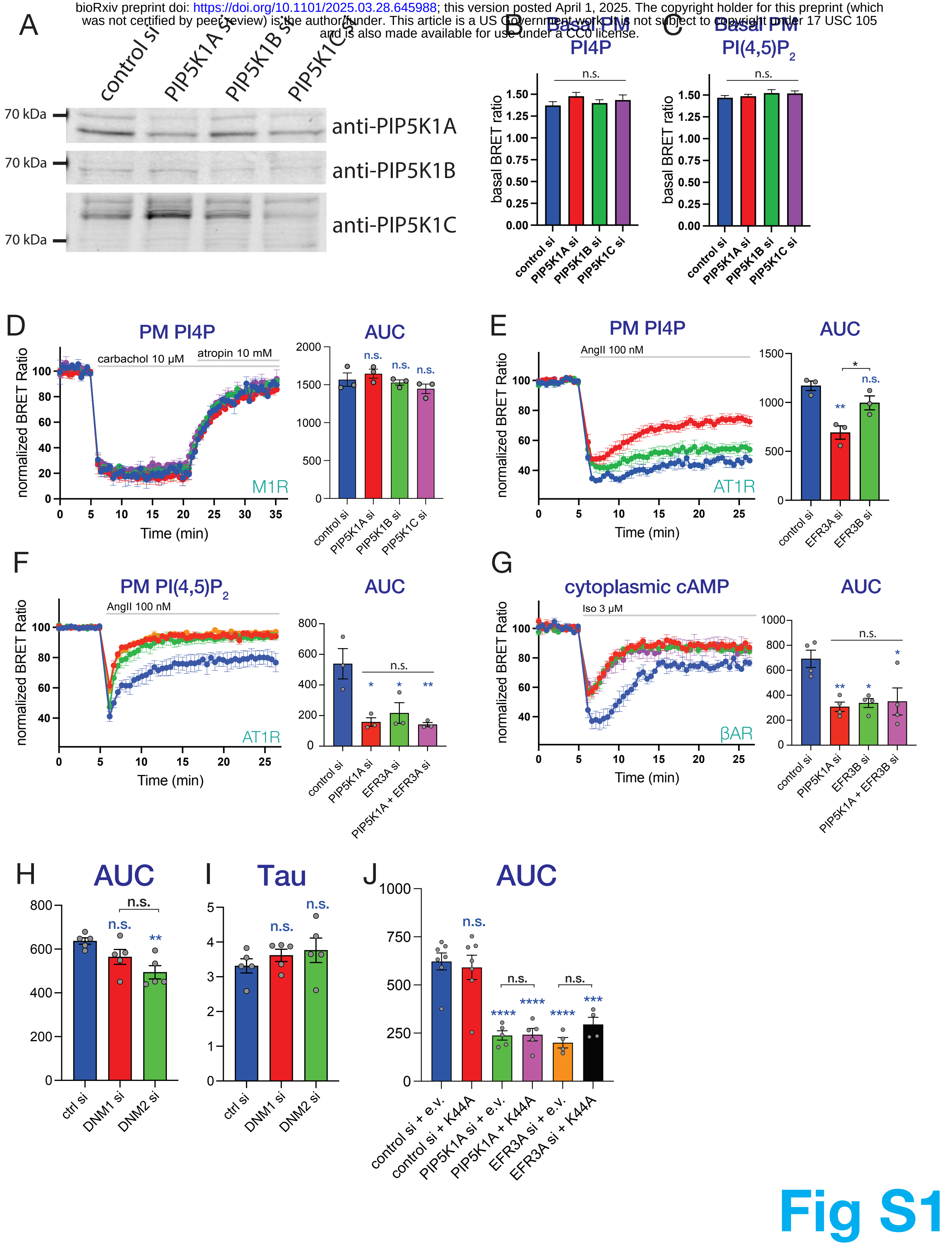

bioRxiv preprint doi: https://doi.org/10.1101/2025.03.28.645988; this version posted April 1, 2025. The copyright holder for this preprint (which was not certified by peer review) is the author/funder. This article is a US Government work. It is not subject to copyright under 17 USC 105 and is also made available for use under a CC0 license.

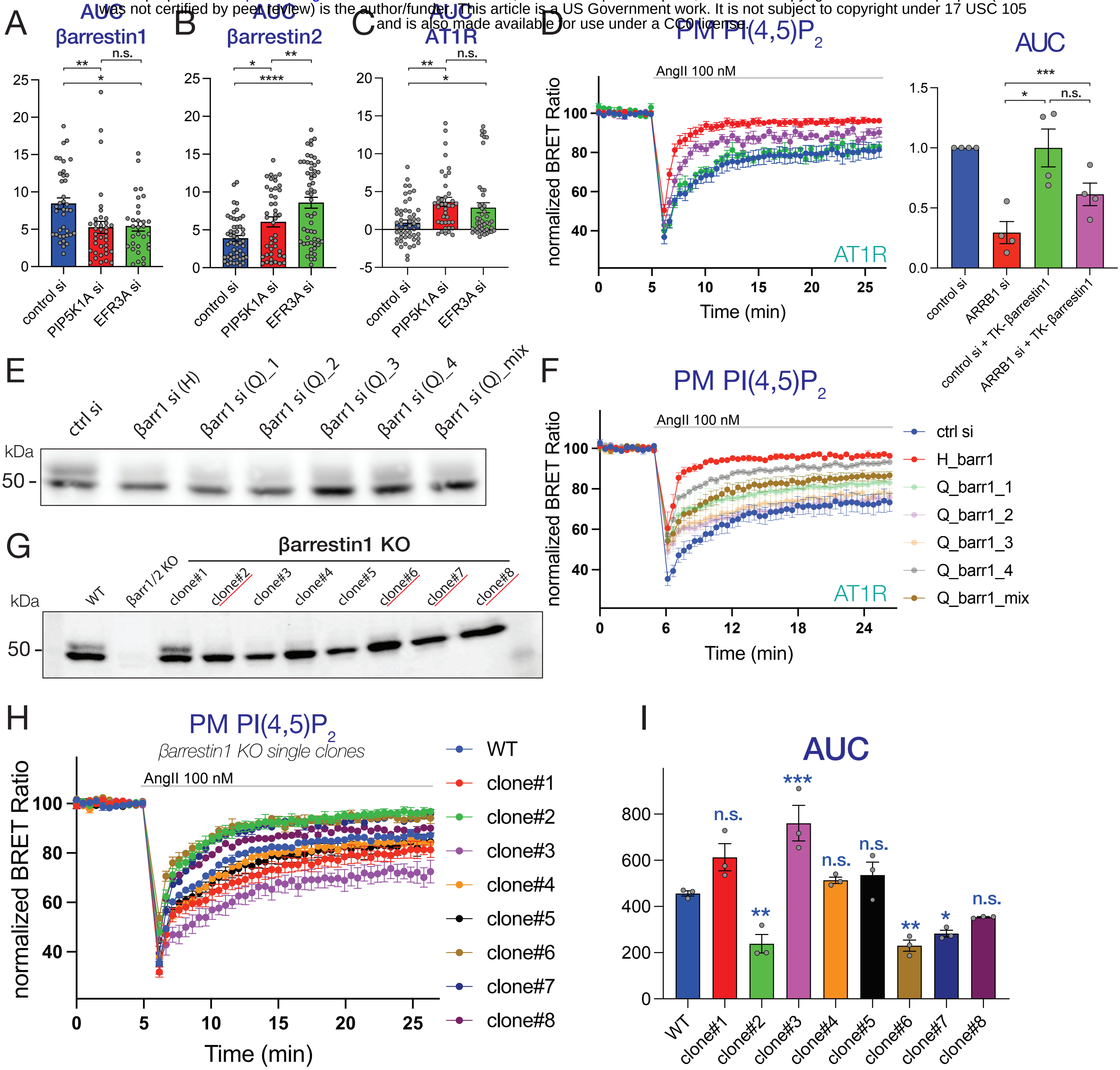

Fig S2

bioRxiv preprint doi: https://doi.org/10.1101/2025.03.28.645988; this version posted April 1, 2025. The copyright holder for this preprint (which was not certified by peer review) is the author/funder. This article is a US Government work. It is not subject to copyright under 17 USC 105 and is also made available for use under a CC0 license.

Fig S3

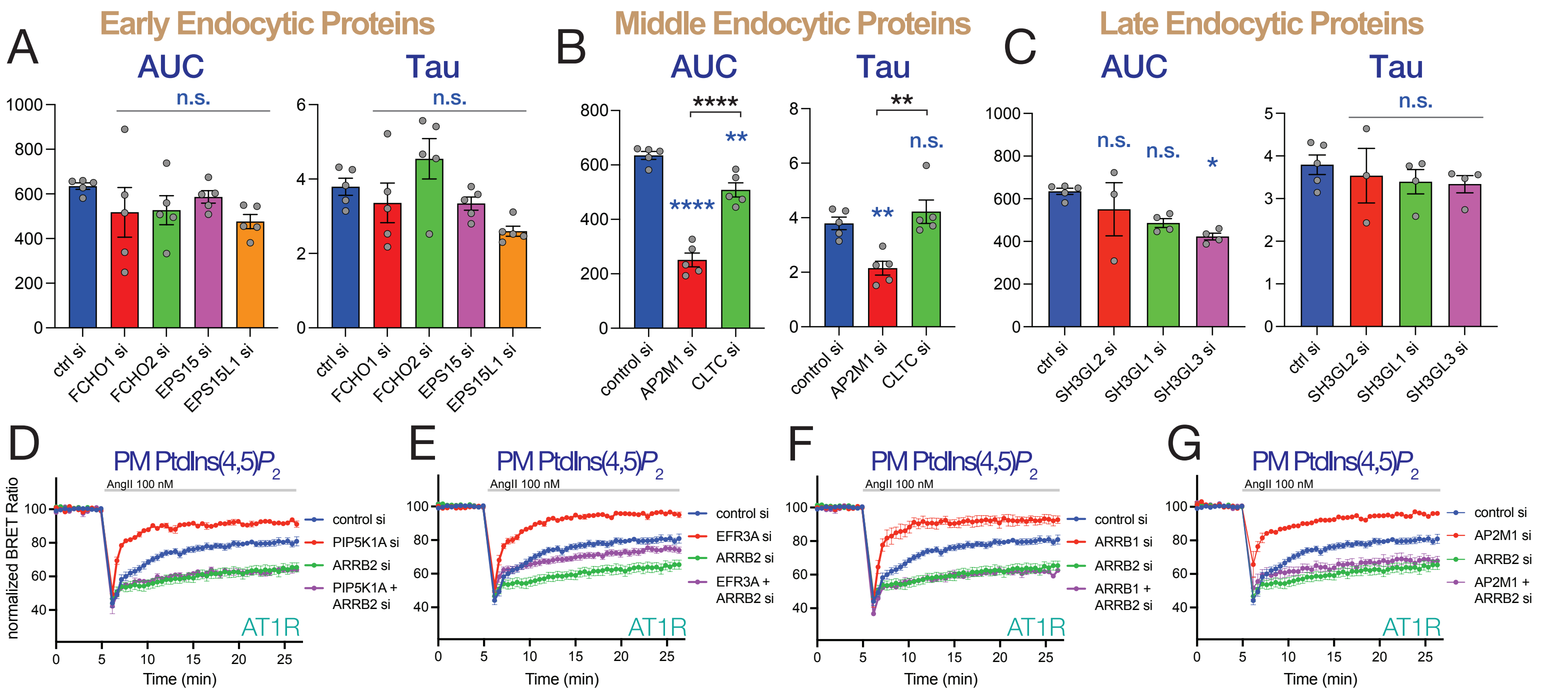

Supplement: Supplement 1 [file NIHPP2025.03.28.645988v1-supplement-1.pdf]
